# A New Look on Protein-Polyphenol Complexation during Honey Storage: Is This a Random or Organized Event with the Help of Dirigent-Like Proteins?

**DOI:** 10.1371/journal.pone.0072897

**Published:** 2013-08-30

**Authors:** Katrina Brudzynski, Calvin Sjaarda, Liset Maldonado-Alvarez

**Affiliations:** 1 Department of Drug Discovery and Development, Bee-Biomedicals Inc., St. Catharines, Ontario, Canada; 2 Department of Biological Sciences, Brock University, St. Catharines, Ontario, Canada; Aligarh Muslim University, India

## Abstract

Honey storage initiates melanoidin formation that involves a cascade of seemingly unguided redox reactions between amino acids/proteins, reducing sugars and polyphenols. In the process, high molecular weight protein-polyphenol complexes are formed, but the mechanism involved remains unknown. The objective of this study was twofold: to determine quantitative and qualitative changes in proteins in honeys stored for prolonged times and in different temperatures and to relate these changes to the formation of protein-polyphenol complexes. Six -month storage decreased the protein content by 46.7% in all tested honeys (t-test, p<0.002) with the rapid reduction occurring during the first three month. The changes in protein levels coincided with alterations in molecular size and net charge of proteins on SDS –PAGE. Electro-blotted proteins reacted with a quinone-specific nitro blue tetrazolium (NBT) on nitrocellulose membranes indicating that quinones derived from oxidized polyphenols formed covalent bonds with proteins. Protein-polyphenol complexes isolated by size-exclusion chromatography differed in size and stoichiometry and fall into two categories: (a) high molecular weight complexes (230–180 kDa) enriched in proteins but possessing a limited reducing activity toward the NBT and (b) lower molecular size complexes (110–85 kDa) enriched in polyphenols but strongly reducing the dye. The variable stoichiometry suggest that the large, “protein-type” complexes were formed by protein cross-linking, while in the smaller, “polyphenol-type” complexes polyphenols were first polymerized prior to protein binding. Quinones preferentially bound a 31 kDa protein which, by the electrospray quadrupole time of flight mass spectrometry (ESI-Qtof-MS) analysis, showed homology to dirigent-like proteins known for assisting in radical coupling and polymerization of phenolic compounds. These findings provide a new look on protein-polyphenol interaction in honey where the reaction of quinones with proteins and polyphenols could possibly be under assumed guidance of dirigent proteins.

## Introduction

Honey processing at high temperatures or its prolonged storage triggers the Maillard reaction that ultimately is responsible for a formation of high molecular weight polymerized brown pigments called melanoidins [1], [2]. According to current views and in vitro models, melanoidin structure and composition result from a condensation and polymerization of nitrogenous and heterocyclic compounds derived from sugar degradation intermediates; deoxyglucosones, Strecker aldehydes, and dicarbonyl compounds [3], [4]. The cascade of these redox reactions lead ultimately to the formation of high molecular weight sugar-derived protein adducts and crosslinks known as melanoidins or advanced glycation end-products (AGEs). While a large number of studies were focused on the protein glycation by dicarbonyl compounds [5]–[7] and sugars in foods subjected to thermal processing [8]–[10], relative fewer studies have documented a polyphenol involvement in protein complexation. Our recent observations showed however, that in addition to sugar-derived protein adducts, honey’s phenolic compounds participated in the melanoidin formation. Progressive binding of polyphenols to proteins and their increased incorporation into brown melanoidins has occurred during honey heating as well as in stored honeys [1], [2], [11]. Functional consequences of melanoidin formation and accumulation included the gradual loss of honey antioxidant and antibacterial activities [11], [12]. These functional impairments’ echoes observations from the earlier studies that showed a causal link between the prolonged storage and a gradual inactivation of two critical honey enzymes: alpha-amylase (diastase) and D-fructofuranoside-fructohydrolase, invertase [13]–[16]. Although the mechanism underlying loss of these functions in honey is not clear, the protein inactivation by complexation with polyphenols could be one of the logical cause worth to investigate.

Polyphenols have the ability to bind proteins forming soluble and insoluble complexes. Protein complexation by polyphenols is a known cause of haze formation in wines, juices and colloids in honey [17]–[19]. The type of interaction, non-covalent or covalent binding between these two components, affects the size and stability of such associations and has both functional and nutritional consequences [20]. In fruits and beverages, the predominant non-covalent interactions between vegetable tannins and proteins occur via a combination of hydrogen bonds and hydrophobic interactions [21]–[23]. Questions remain concerning the type of polyphenol-protein interaction that can be expected in honey with polyphenols of different structures than tannins. Two lines of evidence from our laboratory strongly suggest that the redox capacity of honey polyphenols and the ease with which they can oxidize are prerequisites for a strong, covalent binding to proteins. Firstly, our recent studies on melanoidin structure showed that their size, antioxidant activity and degree of browning directly correlated with the content and radical scavenging activity of bound polyphenols [2], [11]. Secondly, honeys that possessed polyphenols of high radical scavenging activity, such as buckwheat honeys, efficiently produced hydroxyl radicals in the Fenton-like reaction, using as a substrate H_2_O_2_ produced by honey glucose oxidase. This prooxidant activity, in which cytotoxic hydroxyl radicals were generated, was responsible for DNA degradation and inhibition of bacterial growth [24]–[27]. The balance between antioxidant/prooxidant activity in honey can be shifted depending on the external conditions, such as prolonged exposure to oxygen during storage and elevated temperatures. Honey harvesting, pasteurization, and post-harvesting storage are the very conditions that promote polyphenol oxidation, their interaction with proteins and melanoidin formation.

Polyphenol oxidation *in vivo* is usually carried out by polyphenol oxidases; tyrosinases, laccases or peroxidases [28]. However, phenoxyl radicals can also be formed in non-enzymatic reactions via polyphenol auto-oxidation in the presence of oxygen radicals, H_2_O_2_ and metal ions [29], [30]. The phenoxyl radicals (semi-quinones and quinone radicals) can spontaneously react with nucleophilic groups of amino acids and proteins, such as the epsilon amino group of lysine or the sulfhydryl group of cysteine, forming stable, covalent bonds [31], [32]. The examples of protein complexation by quinones relate to melanin formation [33], melanization and sclerotization of insect cuticle [34], [35] and lignin biosynthesis in plants [36]–[38]. This knowledge is therefore vital to improve our understanding of the mechanism and functional consequences of quinone-protein interactions that are relevant not only to honey but also to plant, animal (insect) and human cell biochemistry.

In light of these facts, we hypothesized that honey polyphenols, that can be readily oxidized to quinones, may play a key role in the interaction with honey proteins and that this redox-induced process may underlie the formation of the observed high molecular weight protein-polyphenol complexes.

## Materials and Methods

Honeys were donated by Canadian beekeepers and included both commercial (pasteurized) and apiary (raw) samples. After arrival to laboratory, honeys were assigned an ID number and were tested for color (net absorbance), pH, water activity (Table 1).

**Table 1:** List of honeys.

Honey ID	Botanical origin	Common name	Color (A_560_–A_720_ nm)^*^	pH	Water activity
65	*Cucurbita* spp	pumpkin	0.085	3.5	0.604
66	*Rubus fruticosus*,	blackberry	0.096	3.4	0.608
67	*Vaccinium corymbosum*	blueberry	0.101	3.5	0.586
76	*Fagopyrum esculentum*	buckwheat	0.965	3.6	0.591
77	*Fagopyrum esculentum*	buckwheat	1.886	3.8	0.597
99	*Helianthus annuus*	sunflower	0.298	3.8	0.574
109	*Vaccinium macrocarpon*	cranberry	0.151	3.8	0.546
114	*Helianthus annuus*	sunflower	0.172	3.4	0.56
125	*Fagopyrum esculentum*	buckwheat	1.88	3.8	0.599
177	*Fagopyrum esculentum*	buckwheat	0.66	3.8	0.572
178	*Vaccinium corymbosum*	blueberry	0.165	4.8	0.589
179		wildflower	0.131	4.1	0.558
147	*Leptospermum scoparium*	manuka	0.539	3.9	0.617

### Determination of Protein Concentration

Protein quantification was performed by addition of 200 µL of Bradford dye reagent (brilliant blue G, 0.1 mg/mL; ethanol, 5% (v/v); phosphoric acid, 10% (v/v) and water) to 40 µL of samples, pre-diluted 100 times [39]. The photometrical measure was performed at 595 nm. Bovine serum albumin was used to generate a standard curve. Measurements were done in triplicate.

### Determination of Polyphenol Concentration

The total phenolic content of high molecular weight melanoidins was determined with the Folin-Ciocalteu reagent [40]. Briefly, 790 µL of distilled water, 10 µL of high molecular weight solution, and 50 µL of Folin-Ciocalteu reagent were mixed. After exactly 1 min, 150 µL of 20% aqueous sodium carbonate was added, and the mixture was mixed and allowed to stand at room temperature in the dark for 120 min. Detection was achieved at 760 nm. Gallic acid was used as standard. Measurements were done in triplicate.

### Sodium Dodecyl Sulfate Polyacrylamide Gel Electropheresis (SDS-PAGE)

SDS-PAGE was performed according to the method described by Laemmli, 1970 [41]. Honey proteins were analyzed on 7.5% gel separation gel with attached 5% stacking gel. 30 µl of a honey solution (50% v/v) or 30 µl of a fraction from size-exclusion chromatography were mixed with the sample buffer, denatured at 100°C for 5 min and loaded on a gel. The electrophoresis was carried out in duplicate at a constant current of 100 Volts using a Mini Protean III electrophoresis cell (Bio-Rad Laboratories, Hercules, CA). After electrophoresis, the gel was either stained directly for protein with Coomassie Blue, or the proteins were transferred electrophoretically to nitrocellulose in 25 mM Tris, 192 mM glycine, 0.01% SDS, 20% MeOH for 18 h. The nitrocellulose was stained specifically for quinone-containing proteins with nitro blue tetrazolium in potassium glycinate, as described in Paz et al. [42]. The master gels were fixed in a solution of acetic acid and methanol (1∶1), washed in distilled water and stained with Coomassie Briliant Blue R-250 (Bio-Rad). The molecular weight of the proteins was determined using molecular weight standards (Fermentas Life Sciences, PageRuler Prestained Protein Ladder #SM0671).

### Electroblotting

The proteins were transferred from the SDS-gels into the nitrocellulose membrane using semi-dry system with the anode buffer consisting of 0.3 M Tris, 10% methanol, pH 10.4 and cathode buffer, 25 mM Tris base4, 40 mM glycine, 10% methanol, pH 9.4, at the current density of 1.2 mA/cm^2^ for 1 hour.

### Nitro Blue Tetrazolium Staining

Nitroblue tetrazolium (2-[4-[4-(3,5,-diphenyl-1-H-tetrazol-2-yl}-3-m3thoxy-phenyl]-2-methoxy-phenyl]-3,5-diphenyl-1H-tetrazole) was purchased from Sigma Aldrich (Canada). After electroblotting, nitrocellulose membrane was briefly washed with distilled water and overlaid with the NBT solution (0.6 mg/ml NBT in potassium glycinate buffer, pH 10). The membrane was incubated with shaking for 45 min at room temperature and in the dark. After incubation, the membrane was washed briefly with 0.16 M sodium borate solution, left stand in this solution for 30 min to 1 h and washed with dH_2_O_2_. The photograph was taken after the membrane was air-dried.

### Size-exclusion Chromatography on Sepharose 4B

A Sepharose 4B (Sigma-Aldrich) column (24×1.6 cm) was equilibrated with distilled water at 1 ml/minute prior to use. A 50% (w/v) honey solution in 0.15 M NaCl was centrifuged at 13,000 rpm for 15 min and one milliliter of the SN was loaded onto the column. The fractions (3 mls) were eluted with distilled water (1 ml/min) and monitored at 280 nm. A standard curve for molecular weight determination was generated using a protein kit (Gel Filtration HMW Calibration kit, GE Healthcare) containing ferritin (440 kDa), catalase (240 kDa), aldolase (158 kDa) and albumin (66 kDa). At least 6 separate SEC chromatography was performed on honey H177 at each storage temperature (−20°C, 4°C and room temperature).

### In-Gel Tryptic Digestion

Bands were excised from Coomassie blue-stained gels, cut into 1-mm cubes, and washed in distilled water followed by acetonitrile:100 mM NH_4_HCO_3_ solutions, as described in Protocols, the Biological Mass Spectrometry Laboratory, UWO (http://www.bmsl.uwo.ca/in-gel_digestion.html). Proteins were reduced with dithiothreitol, followed by alkylation with iodoacetamide to prevent free thiol groups from cross-linking. Gel bands were dried in acetonitrile and rehydrated in ammonium bicarbonate. Proteins in the gel were digested by incubation with nonself cleaving trypsin (Promega, Madison, WI) for 16 h at 37°C, extracted with a acetonitrile/sodium bicarbonate solution and dried in a speed vacuum. Prior to mass spectrometric analysis, dried peptide samples were re-dissolved in a 0.2% formic acid and 3 ul of volumes were injected into mass spectrometer.

### ESI-Q-TOF-MS/MS Analysis

ESI-Q-TOF- MS/MS analyses (electrospray quadrupole time of flight mass spectrometry) were performed using a Q-TOF Global mass spectrometer (Waters) and run in positive mode using electrospray interface. MS acquisition was obtained with MassLynx 4.1 software (Waters). The columns consisted of Waters nanoAcquity UPLC column (BEH130 C18) with a trap column (Symmetry C18). Peptides were resolved at 300 nl/min over a 75 min in a gradient of water (A) and 0.2% formic acid (B): methanol.

Time (min)  %A     %B.

Initial     95     5.

40.0     40     60.

42.5     5    95

47.5     5     95

50.0     95     5

75.0     95     5

Mass spectra of peptides were acquired over the mass range 300–1800 m/z with the capillary voltage set at 1900 V, collision energy set at 10 V, and sample cone set at 50 V for the entire 75-min gradient.

Peptide mapping and database analyses were conducted using Mascot (www.matrixscience.com) software.

### Statistical Analysis

Analyses were performed using the statistical program GraphPad Instat version 3.05. (GraphPad Software Inc.). Data were analyzed using a one-way ANOVA with subsequent Tukey-Kramer Multiple Comparison test or an unpaired t-test. Differences between means were considered to be significant at p<0.05.

## Results and Discussion

### Qualitative Changes in the Protein Content during Honey Storage

As the first step toward elucidating the relationship between protein content, protein-polyphenol interaction and complexation and storage time, we monitored changes in honey proteins at quantitative (Bradford assay) and qualitative levels (SDS-PAGE). The comparison of protein concentrations between different honeys stored under the same conditions for the same period of 6 months showed that storage led to a significant decrease in protein content with an average decrease of 46.7% in all tested honeys (t-test, n = 24, p<0.002) (Table 2). The decline was a common occurrence during storage independently of honey botanical origin/chemical composition or the initial protein concentrations (Table 2).

**Table 2:** Changes in the protein concentration in different honeys during storage (n = 3–6).

Honey	Total proteinconcentrations (µg/ml)	Total protein concentrations after6 month storage at RT (µg/ml)	Percentage of decrease
Buckwheat H77	3095.7±4.66	1941±5.76	37.30%
Buckwheat H76	2376.8±3.89	1455.8±6.9	38.70%
Buckwheat H177	1465.7±1.79	623.15±3.21	57.50%
Sunflower H99	1950.2±2.02	852.2±2.88	56.30%
Sunflower H114	1375.9±6.43	583.9±0.79	57.50%
Cranberry H109	1296.3±3.33	616.3±1.01	52.45%
Blueberry H178	453.9±1.12	156.5±0.9	26%
Wildflower H179	289.3±0.99	96.2±0.1	47.70%

**Figure 1:**
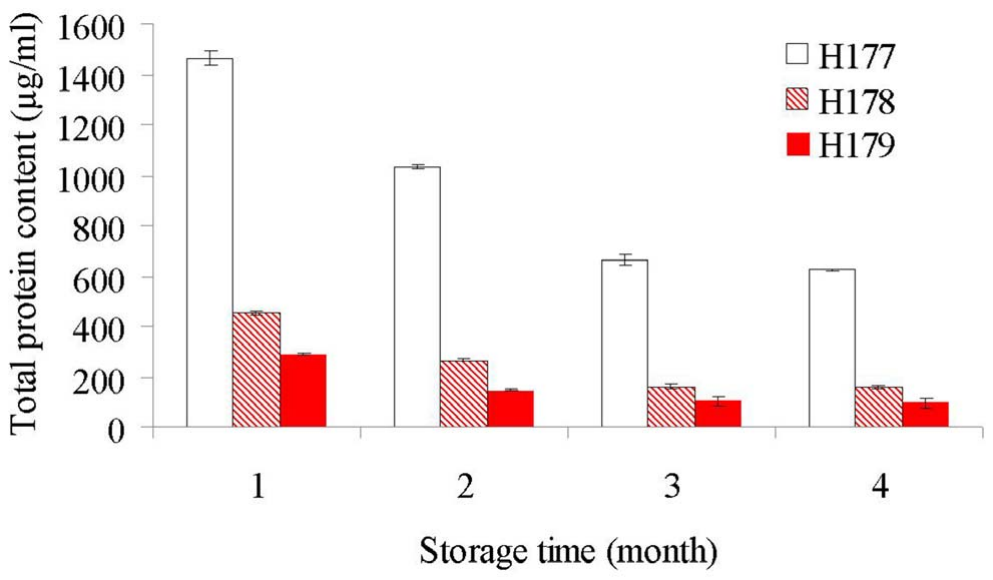
Changes in protein concentrations during the first 3-month storage time of honey.

To follow kinetics of changes in protein concentrations as a function of storage time, we have randomly chosen three honeys from our pool of honeys (buckwheat honey H177, blueberry honey H178 and wildflower honey H179) and analyzed their protein content in monthly intervals over a period of four month. The graphic presentation of a concentration change showed a relatively rapid decrease in the total protein content during first three month of storage (Fig. 1). Thereafter the rate of decrease was slower finally reaching a plateau at which the mean values of protein content were not significantly different (Fig. 1). Although the protein concentrations varied between tested honeys, the changes over storage time followed a similar trend.

### Modification of Honey Proteins by Quinones

**Figure 2:**
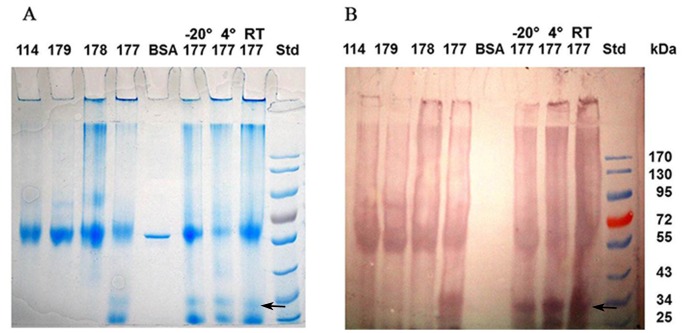
Visualization of quinone-protein adducts by using a quione-specific nitro blue tetrazolium staining. The Coomassie blue-stained gel of proteins of different honeys and that of buckwheat honey (H177) stored at room temperature (RT), 4°C and −20°C (**A**) and its corresponding electroblot stained with the quinone-specific nitro blue tatrazolium (**B**) BSA was used as a control for quinone-staining specificity. The arrow indicates a position of the 31 kDa protein.

We hypothesized that honey polyphenols that can be readily oxidized to quinones, may play a key role in the interaction with honey proteins and their complexation. To explore this possibility, we sought evidence for the presence of quinones in protein bands resolved in SDS-PAGE. The proteins of honey samples were separated using SDS-PAGE, electroblotted to a nitrocellulose membrane and then probed with nitro blue tetrazolium (NBT) in a glycinate buffer (pH 10), that is, under alkaline conditions that allow a specific reaction with quinones [42]. Figure 2 shows that indeed quinone-protein adducts were formed in stored honeys. The NBT staining was specific for honey proteins naturally exposed to polyphenols, as opposed to the control proteins such as BSA which was not exposed to polyphenols and subsequently did not react with the dye (Fig. 2).

**Figure 3:**
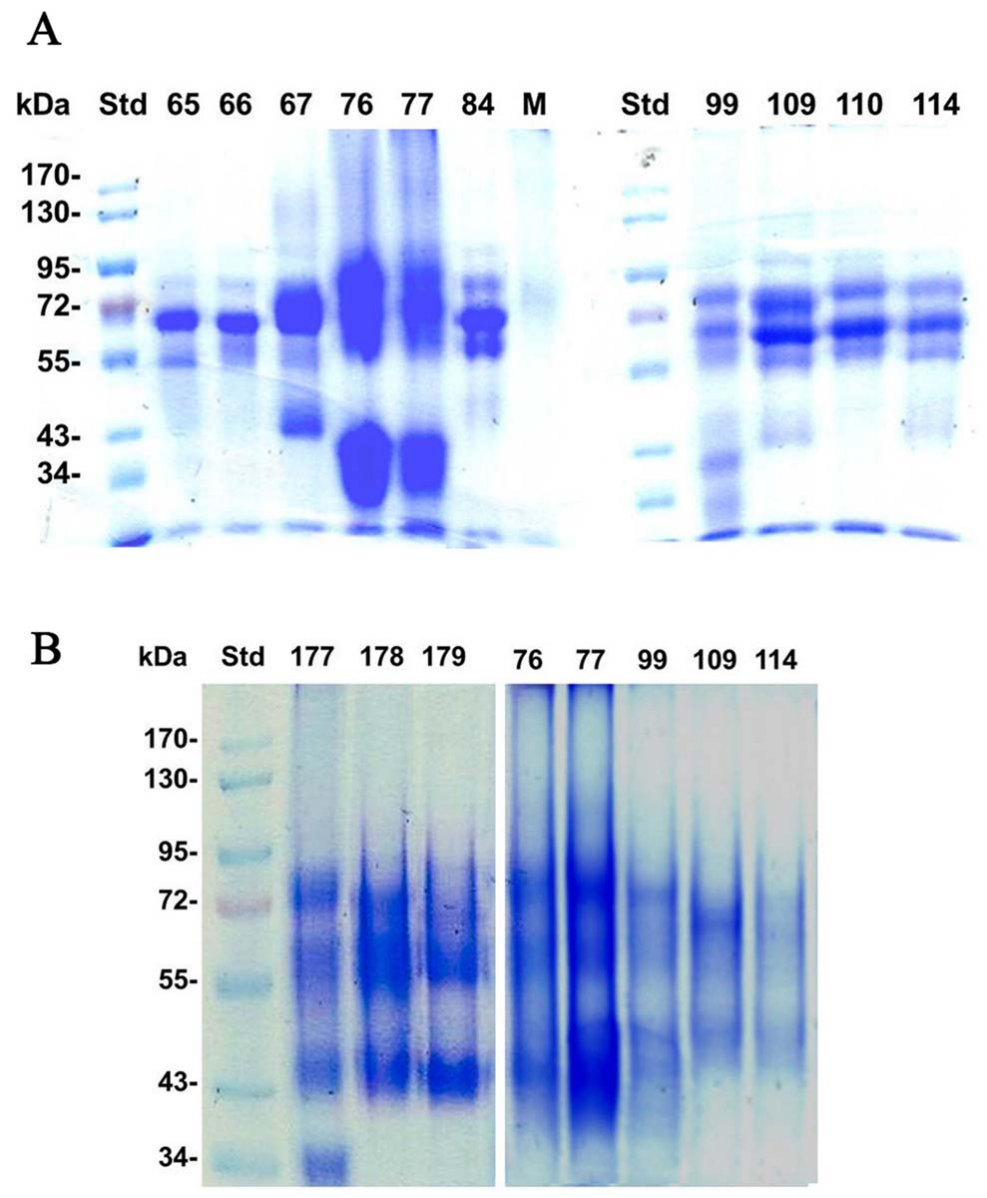
Changes in protein profiles and electrophoretic mobility of proteins induced by 3- month storage of honeys at room temperature. (**A**) fresh honeys; (**B**) stored honeys.

Quinone binding to proteins was intensified by honey storage at elevated temperatures and led to protein modifications that changed their net charge and size (Fig. 2B). The modified proteins showed an altered electrophoretic mobility during SDS-PAGE, produced diffuse band appearance and led to the reduction of protein content in the bands (Fig. 3B versus 3A) or a complete band disappearance (as in honeys 99, 109 or 114, Fig. 3B versus 3A). The latter observations suggested that quinone- modified proteins become less soluble and formed complexes that were either too big to enter the gel [2] or simply insoluble, creating an impression of band disappearance. However, direct evidence for the formation of protein-polyphenols complexes came from size-exclusion chromatography.

### Size Exclusion Chromatography

**Figure 4:**
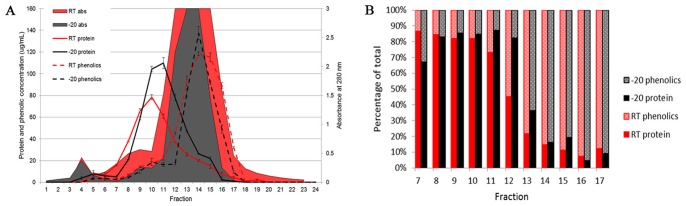
Comparative distribution and quantitation of proteins and polyphenols in protein-polyphenol complexes, A SEC profile (at A 280 nm) of the size distribution of protein-polyphenol complexes in buckwheat honey (H177) stored at −20°C (black line) and at room temperature (red line), and the distribution of proteins (as estimated by Bradford method, black line, −20°C, red line, RT) and polyphenols (as estimated by Folin-Ciocalteu method, black dotted line, −20°C and red dotted line, RT) (**A**). The protein to polyphenol ratio in fractions expressed as percentage of the total mass (**B**).

Size-exclusion chromatography of buckwheat honey (H177) allowed the separation of protein-polyphenol complexes ranging from over 200 to 85 kDa (Fig. 4, Table 3). The isolated fractions showed differences in protein to polyphenol ratios. In high molecular weight complexes (230–180 kDa) the protein content amounted up to 80% of total mass (fraction F12) while the opposite proportion was observed in lower molecular weight complexes (110–85 kDa) where polyphenol content amounted up to 90% of total mass (fraction F14, Fig. 4 and Table 4). Considering the average size of a polyphenol as 0.5 kDa and the size of the most abundant protein in these fractions as 56 kDa, the stoichiometry would amount to 1∶137 and 1∶656 protein/polyphenol molar ratio for complexes in fraction F12 and F14, respectively. At low polyphenol/protein ratios, the size of formed complexes (∼200 kDa) suggested that polyphenols bound and cross-linked adjacent protein chains. The occurrence of such cross-links has been observed in model systems where oxidized phenolics reacted with α-lactalbumin, lysozyme and BSA [43] or RNase [44].

**Table 3:** Molecular size of fractions eluted from SEC.

Honey fraction	Molecular size (kDa)
13	180–232
14	140–180
15	109–140
16	85–109

**Table 4:** The ratio of protein to polyphenols in SEC fractions (n = 9).

Fraction	Protein conc. (mg/ml)		Phenolics conc. (mg/ml)		Ratio (RT)		Ratio (−20°C)	
	RT	−20°C	RT	−20°C	p/pp*	pp/p**	p/pp	pp/p
7	15.89±0.92	5.10±1.47	2.49±0.63	2.49±0.63	6.38	0.15	2.04	0.48
8	37.72±0.51	20.87±0.06	6.89±0.63	4.30±0.637	5.47	0.18	4.85	0.2
9	65.83±1.94	58.95±1.41	14.4±0.62	10.02±1.65	4.57	0.21	5.88	0.16
10	78.41±2.27	104.27±2.57	17.29±1.72	18.70±3.25	4.53	0.22	5.57	0.17
11	60.97±2.02	109.72±5.11	22.19±1.72	16.28±1.65	2.74	0.36	6.74	0.15
12	36.89±0.06	77.93±2.87	45.11±1.08	16.9±1.09	0.81	1.22	4.61	0.21
13	25.74±1.26	47.09±0.89	93.3±4.48	82.7±3.25	0.27	3.62	0.56	1.75
14	20.16±0.89	26.21±0.41	118.19±1.79	137.34±5.99	0.17	5.86	0.19	5.23
15	14.59±1.68	21.94±1.42	115.21±3.97	91.74±1.55	0.12	7.89	0.23	4.18
16	7.12±2.26	2.37±0.83	89.13±2.76	48.87±3.76	0.08	12.5	0.048	20.6

* p/pp = protein to polyphenol ratio.

** pp/p = polyphenol to protein ratio.

At low protein/polyphenol ratio, the complexes were of smaller sizes (110–85 kDa) and predominantly built of polyphenols (Fig. 4 and Table 4). Studies on interactions of tannins with proteins suggested that at low protein/polyphenol ratios proteins become “coated” by polyphenols as a result of non-specific, non-covalent, mostly hydrophobic interactions [20]–[23]. In contrast, under the same conditions, quinone radicals formed covalent bonds with both proteins and other phenolics. Quinone-quinone interactions preceded protein binding and led to the formation of dimeric and higher molecular weight condensation products [23], [45]. Moreover, polymerized phenolics were found to be more reactive toward proteins [46]. The latter data are in agreement with the type of interaction observed in complexes formed at low protein/polyphenol ratios in our study. Firstly, the observation those protein-polyphenol complexes eluted as a single entity during size-exclusion chromatography points to the formation of stable, covalent bonds between these two components. Secondly, the size of complexes (about 100 kDa) compared with the average size of a polyphenol monomer (0.5 kDa) add to the argument that at low protein/polyphenol ratios, polyphenol polymerization must occur prior to protein binding. Thirdly, our most recent LC- ESI/MS analysis of these complexes indeed showed the presence of esters and dimers of ferulic, caffeic, quinic and *p*-coumaric acids (data not shown). The radical coupling of hydroxycinnamates and their oligomers have been shown to participate in lignin binding and their cross-linking with polysaccharides of plant cell wall [36] but have never been shown in honey. Thus, our results suggest new types of protein-polyphenol complexes in honey that are formed due to polyphenol oxidation to quinones.

### Enhanced Quinone Binding to the 31 kDa

**Figure 5:**
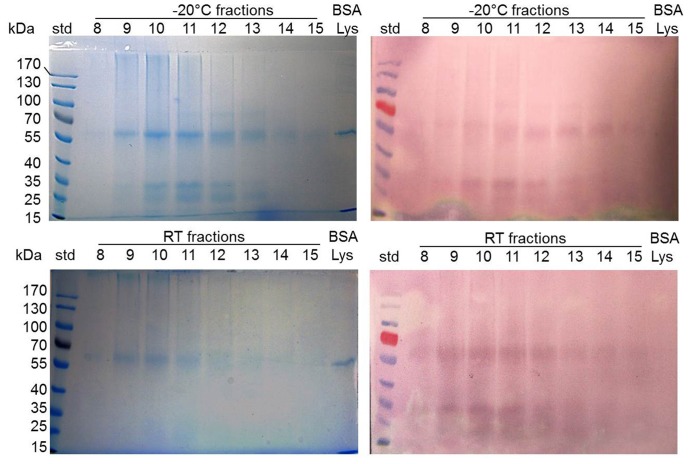
Enhaced quinone binding by the 31 kDa honey protein. Quinone-containing proteins in SEC fractions of honey stored at −20°C and at room temp representing Coomassie blue-stained proteins of the master SDS gel and (left panel) corresponding blot of NBT-positive proteins (right panel). BSA and lysozyme were used as a control.

The quinone-protein adduct formation depended on a nature of the protein and the temperature at which honey was stored (−20°C versus room temperature). We found that the intensity of NBT staining of the proteins on the blots did not always correspond to the abundance of a protein band seen in the Coomassie blue stained SDS gels. When the same volume (30 µl) of fractions from the size-exclusion chromatography was applied to the gel, the intensity of the NBT staining of a 31 kDa protein band was disproportionately high relative to their staining by Coomassie blue on gels (Fig. 5). The storage temperature of honeys (−20°C versus room temperature) reduced the content of soluble proteins, specifically, the 31 kDa protein (Fig. 5). Thus, the 31 kDa protein of buckwheat honey seemed to be a preferential target for quinone binding.

### Enhanced Quinone Binding to the 31 kDa Protein during Honey Storage (Aging)

Polyphenol oxidation and radical coupling show a strong dependence on the temperature. To accelerate the oxidation processes that take place during honey storage, mimicking the “aging” of honey, aliquots of honey H177 were incubated at room temperature (RT), 37°, 57° and 77°C for 4 hours. The purpose of the accelerated-aging tests was to provide experimental data in support of our notion that the 31 kDa protein was a target molecule for quinone binding and that this binding was enhanced with honey “aging”.

As in previous experiments, the changes in the protein levels during aging were estimated from the amount of Coomassie blue staining of SDS gels, while the changes in quinone-protein interactions were estimated from the levels of NBT staining of the corresponding blots. In both cases, the differences in the amounts of stain retained during aging were presented in semi-quantitative way using Bio-Rad Imaging System (VersaDoc 4000 MP)(Bio-Rad Image Lab 4.1, Hercules, CA).

**Figure 6:**
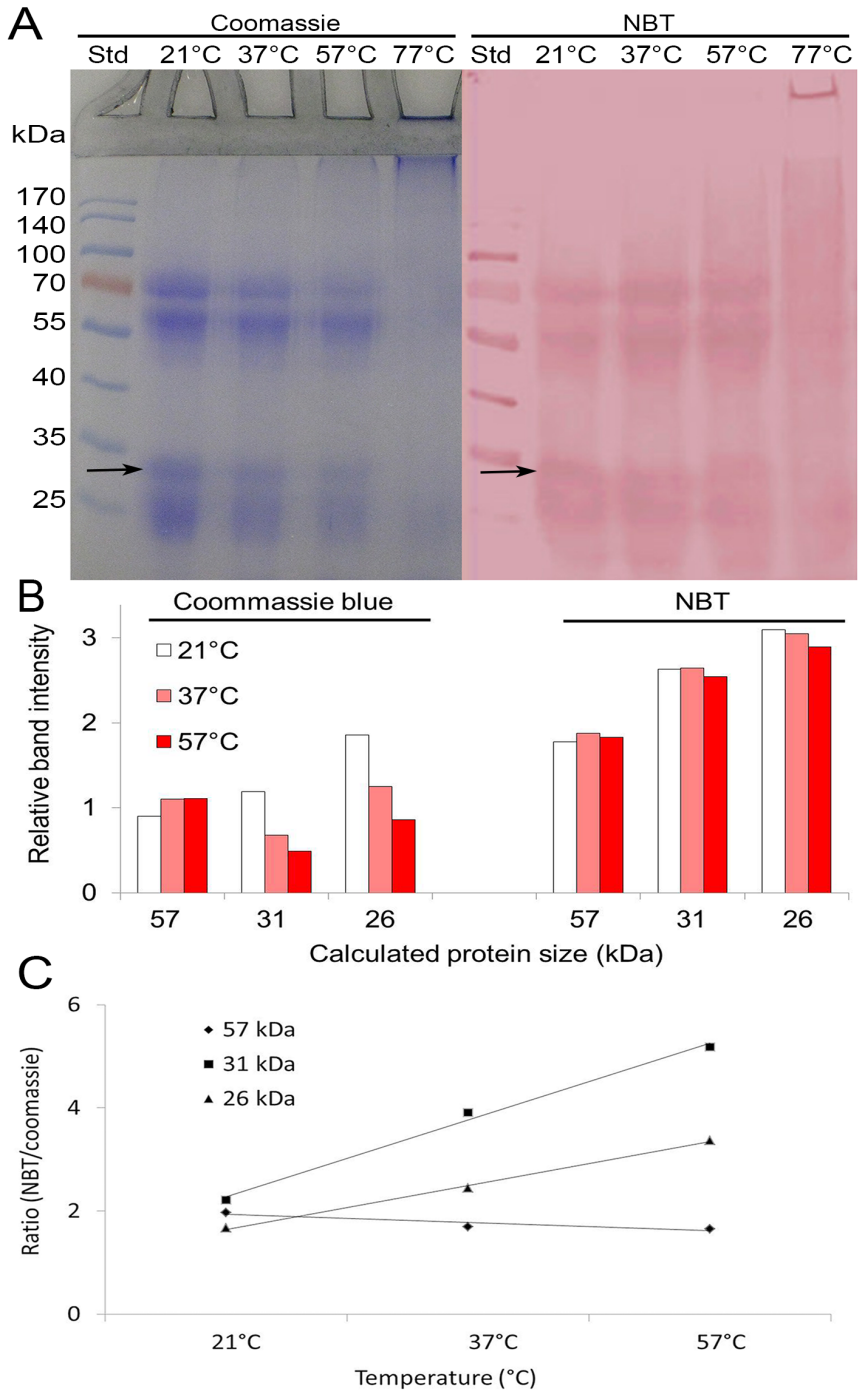
Quinone binding by 31 kDa protein is temperature-dependent. The effect of the accelerated aging of honey H177 on the content of the 31 kDa protein as evidenced by the Coomassie blue-stained SDS gels and on its interaction with quinones, as evidenced by the NBT staining of the blots (A). The arrow indicates a position of the 31 kDa protein. A semi-quantitative representation of these changes in the Coomassie stained SDS gels and in the NBT stained blots (B). The aging -dependent increase in quinone- 31 kDa protein binding visualized as a relative ratio of the NBT/Coomassie staining (C).

Figure 6 shows that not all of the honey’s proteins in SDS gels were equally sensitive to the accelerated aging. The Coomassie blue staining of the 57 kDa protein remained relatively unchanged over the temperature range (RT to 57°C) compared with the marked reduction of staining of the 31 kDa and 26 kDa proteins. In contrast, there was only a slight decrease in the NBT staining of the latter proteins on the blots (Fig. 6A and B). The apparently unchanged kinetics of the quinone-protein binding (the NBT staining) compared to the relatively rapid rate of decrease in the protein content (the Coomassie staining) suggested the aging-dependent, enhanced interaction between the 31 kDa and 26 kDa proteins and quinones. This trend was better visualized by the graph showing the increased ratio of the retained NBT to Coomassie staining for the 31 kDa and 26 kDa proteins while the ratio for the 57 kDa protein remained only slightly altered (Fig. 6C). Thus, the 31 and 26 kDa proteins were the most prominent proteins in the buckwheat honey sensitive to storage time.

### ESI-MS/MS Analysis of 31 kDa

In order to determine the nature of the 31 kDa protein, the band excised from the gels was subjected to in-gel tryptic digestion and analyzed by ESI-Q-TOF- MS/MS (electrospray quadrupole time of flight mass spectrometry). Subsequent Mascot database search using the peptide mass fingerprints produced a statistically significant score of 195 (where scores >46 indicated identity or extensive homology, p<0.05) with taxonomic restriction to green plants (Viridiplantae). The proteins matching the same 3 sets of peptides were identified as a dirigent-related protein (*Tamarix androssowii*) (Accession number: gi|92122701) a putative disease resistance response protein (*Ricinus communis*) (Accession number: gi|255574738) (score 194) and a hypothetical protein (*Vitis vinifera*) (Accession number: gi|225427957) (score 194) (Table 5 and 6).

**Table 5:** Peptide sequence report summary.

Query	Experimental relativemolecular mass	Calculated relativemolecular mass	Mascot Score	Peptide
14	987.517	987.5502	59	LPVVGGTGVFRM
20	1100.6208	1100.6343	57	ELPVVGGTGVFRM
28	1229.657	1229.6768	79	RELPVVGGTGVFRM

**Table 6:** Proteins sharing homology with sequenced peptide.

Protein name	Accession Number	Coverage (%)	Mascot score
Dirigent-related protein: *Tamarix androssowii*	gi|92122701	7.6	195
Disease- resistance response protein, putative: *Ricinus communis*	gi|255574738	7.5	194
Hypothetical protein: *Vitis vinifera*	gi|225427957	7.6	194

Dirigent proteins are relatively recent discovery of Davin et al. (1997) [38]. They are considered to play a role in directing the stereoselective phenoxy radical coupling reaction of two molecules of coniferyl alcohols in lignin synthesis in plants. They do not have enzymatic or redox function by themselves but they control the order of polymerization of phenoxyl radicals [38]. Some dirigent proteins have been shown to be involved in the plant disease-resistance response [47]. Expression of dirigent genes in plants such as spruce *(Picea* spp*)* is induced during disease-resistant response suggesting that dirigent- and disease resistance proteins may both act together as a part of plant defence system. Dirigent protein-guided free radical coupling of monolignols in lignin synthesis serves to tighten the plant cell wall in order to protect the plant against pathogens or invaders [48].

**Figure 7:**
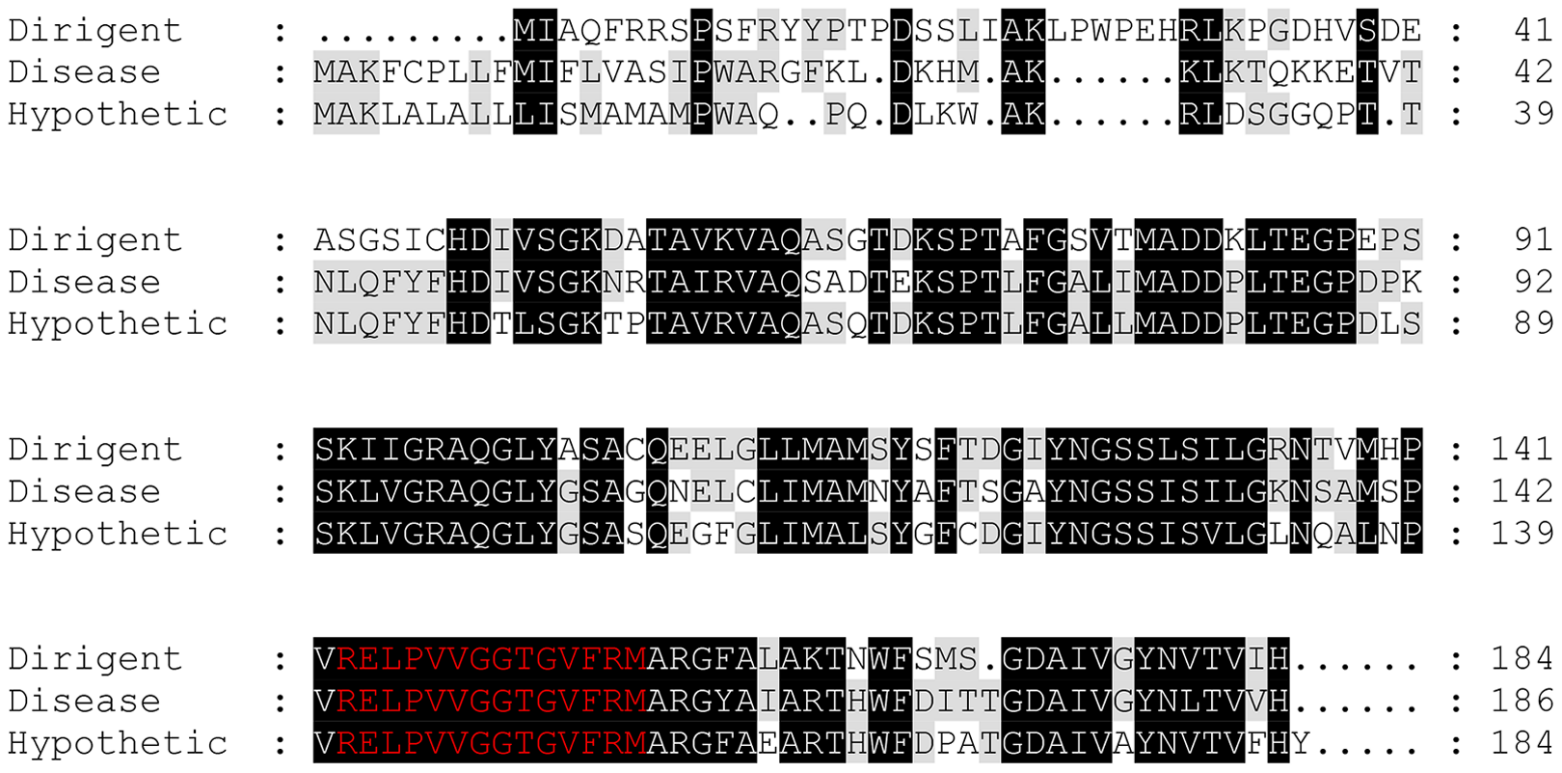
Sequence homologies. A sequence alignment of the dirigent- related protein (*Tamarix androssowii*), disease-response protein of *Ricinus communis* and the hypothetical disease-resistance protein of *Vitis vinifera* showing regions of similarity (black highlight), including the conserved domain which indicate 100% homology to the 31 kDa protein found in honey (red letters).

Indeed, the sequence alignment shows significant similarity of the dirigent-like protein from *Tamarix androssowii* (gi|92122701) with disease-response protein of *Ricinus communis* (gi|255574738) and the hypothetical disease-resistance protein of *Vitis vinifera* (gi|225427957) (Fig. 7). NCBI BLAST search for the conserved domain (www.ncbi.nlm.nih.gov 〉 NCBI 〉 Domains & Structures) revealed that the 14 amino- acid sequence motif (red letters) that is present in the 31 kDa protein corresponds to a conserved domain of dirigent superfamily of proteins. One inference from this observation is that the 31 kDa protein may play similar biochemical function with dirigent and diseases-response proteins by facilitating polyphenol polymerization. Consequently, it might have a specific affinity for a phenolic substrate as indicated by the marked accumulation of the NBT staining (Fig. 2 and 5).

Recent data suggest that the function of dirigent proteins might not be restricted only to lignin biosynthesis but could be extended to controlling the quinone-based polymer formation during insect cuticle melanization and sclerotization [34], [35]. Only a handful of dirigent proteins have been identified so far, most *in planta*. Honey is a unique mixture of chemical compounds of honeybee- and plant-origin where polyphenols and sugars from plant nectars meet proteins from pollen and from the secretion of honeybee hypopharyngeal glands. For the most part, the interactions between these molecules and the formation of protein-polyphenol-sugar complexes were considered a random event that resulted from the diffusion and hit and miss interactions. In this context, discovery of dirigent-like proteins in honey may suggest a novel organizational mechanism for assembly of polymeric complexes. This exciting possibility requires further studies.

The biological significance of quinone-protein and quinone-quinone interactions extends far beyond the phenomena observed in honey. Quinones are common compounds of prokaryotic and eukaryotic cells arising from the enzymatic and non-enzymatic oxidation of polyphenolic precursors. Their covalent associations with cellular macromolecules have been shown to play a key role in plant, fungal and animal pigmentation, insect cuticle formation and lignin biosynthesis in plants. The quinone-protein complexation occurs to a significant extent during food processing. Our results therefore allow a better understanding of the connection between redox-active phenolics, phenolic oligomerization and protein binding and the diverse functional consequences these interactions may have for food and biological systems.

In conclusion, we provided evidence that honey polyphenols interact with proteins and can form high molecular weight complexes. The key molecules engaged in the interactions with proteins were quinones. Quinones produced via auto-oxidation formed stable, covalent bonds with proteins and polyphenols. Unravelling the presence of dirigent –like proteins in buckwheat honey suggests that they may play role in organized assembly of these complexes.
